# Women’s mood at high altitude. sexual dimorphism in hypoxic stress modulation by the tryptophan–melatonin axis

**DOI:** 10.3389/fphys.2022.1099276

**Published:** 2023-01-17

**Authors:** D. E. Alcantara-Zapata, N. Lucero, N. De Gregorio, P. Astudillo Cornejo, C. Ibarra Villanueva, M. J. Baltodano-Calle, G. F. Gonzales, C. Behn

**Affiliations:** ^1^ Laboratorio de Endocrinología y Reproducción, Laboratorios de Investigación y Desarrollo (LID), Facultad de Ciencias y Filosofía, Universidad Peruana Cayetano Heredia, Lima, Perú; ^2^ Occupational Health Program, School of Public Health, University of Chile, Santiago, Chile; ^3^ Laboratory of Extreme Environments, Department of Physiology and Biophysics, Biomedical Science Institute (ICBM), Faculty of Medicine, University of Chile, Santiago, Chile; ^4^ Occupational Ergonomics Program, Department of Kinesiology, University of Atacama, Copiapó, Chile; ^5^ High Altitude Research Institute, Universidad Peruana Cayetano Heredia, Lima, Perú; ^6^ Faculty of Medicine, University of Atacama, Copiapó, Chile

**Keywords:** women’s mood, high altitude, serotonin, tryptophan, REM sleep, thermogenesis

## Abstract

Sexual (and gender)-dimorphism in tolerance to hypobaric hypoxia increasingly matters for a differential surveillance of human activities at high altitude (HA). At low altitudes, the prevalence of anxiety and depression in women has already been found to double when compared with men; it could be expected to even increase on exposure to HA. In purposefully caring for the health of women at HA, the present work explores the potential involvement of the tryptophan (Trp)–melatonin axis in mood changes on exposure to hypobaric hypoxia. The present work highlights some already known anxiogenic effects of HA exposure. Hypoxia and insomnia reduce serotonin (5-HT) availability; the latter defect being expressed as failure of brown adipose tissue (BAT) activation and mood disorders. Rapid eye movement (REM) sleep organization and synapsis restoration that are additionally affected by hypoxia impair memory consolidation. Affective complaints may thus surge, evolving into anxiety and depression. Sex-related differences in neural network organization and hormonal changes during the menstrual cycle, and certainly also during the life cycle, underscore the possibility of 5-HT–related mood alterations, particularly in women on HA exposure. The mean brain rate of 5-HT synthesis at sea level is already 1.5-fold higher in males than in females. sexual dimorphism also evidences the overexpression effects of SERT, a 5-HT transporter protein. Gonadal and thyroid hormones, as influenced by HA exposure, further modulate 5-HT availability and its effects in women. Besides caring for adequate oxygenation and maintenance of one’s body core temperature, special precautions concerning women sojourning at HA should include close observations of hormonal cycles and, perhaps, also trials with targeted antidepressants.

## 1 Introduction

One’s mood tends to change on high altitude (HA) exposure (Wang et al., 2014; Kanekar et al., 2015; Das et al., 2018; Kious et al., 2018; Reno et al., 2018). HA exposure may even provoke suicide (Young, 2013; Bocchetta and Traccis, 2017), with various factors possibly involved (Reno et al., 2018). In fact, atmospheric pressure and completed suicide rates evidence a highly significant inverse correlation (Frutos et al., 2018). Mood changes, the quality of life, and cognitive performance are, however, greatly improved by adequate oxygenation in hypoxic patients (Krop et al., 1973). Women appear to be more susceptible to acute mountain sickness than do men (Richalet et al., 2012; Richalet et al., 2020; Canouï-Poitrine et al., 2014). On exposure to HA, peripheral edema also occurs more frequently in women than in men (Canouï-Poitrine et al., 2014; Richalet et al., 2020). Rotating night shift work, mostly implicit to working at HA, notably increases the risk of ischemic stroke, again particularly in women (Brown et al., 2009). Moreover, night shift work that represents a potential carcinogenetic condition has also been especially proven in women (Gehlert and Clanton, 2020).

Female rats were shown to be more prone to mental alterations on exposure to HA than their male counterparts (Kanekar et al., 2015). Reproductive issues have been shown to change on exposure to HA (Gonzales and Carrillo, 1993; Escudero et al., 1996; Gonzales et al., 2002); puberty, menstrual cycle, pregnancy, and menopause are all known to modulate the onset, recurrence, and exacerbation of affective disorders (Altemus et al., 2014). Oral contraception, menopause, and hormonal treatment do not appear to influence HA effects (Richalet et al., 2020). Sleep changes, however, occur during the menstrual cycle (Baker and Lee, 2018). Being a woman features indeed among the independent predictors of significant anxiety at HA (Boos et al., 2018). Anxiety levels and even somatic alterations at HA correlate with pre-expedition propensity (Boos et al., 2018). However, sexual (and gender)-dimorphism, referring to mood changes on exposure to HA, remains to be more closely investigated; specifically, the serotonin (5-HT) system that constitutes a part of the tryptophan (Trp)–melatonin axis and is known for many sex-related differences (Pitychoutis et al., 2012). 5-HT is involved in sleep regulation and mood expression, and additionally influences appetite, gastrointestinal motility, sexual behavior, pain, and certainly also thermoregulation.

HA exposure alters the circadian rhythm of melatonin that is often related to energy metabolism (Behn and De Gregorio, 2020). This finding prompted us to explore the Trp–melatonin axis, as a field full of neurochemically active and anxiogenic derivatives, strongly linked to sex hormones, inflammation, and energy metabolism (Cipolla-Neto et al., 2022). Hypoxia-induced decreases in energy metabolism affect thermoregulation, the latter being further impaired by the failure of 5-HT–dependent brown adipose tissue (BAT) activation. HA-related insomnia additionally interferes with thermoregulation and probably also with sleep-related neural circuitry restoration. Memory consolidation reduced by inadequate REM sleep predisposes one to anxiety and depression. Affective disorders impair the safety and productivity at work (Matamala Pizarro and Aguayo Fuenzalida, 2021). The main topics discussed here are potential HA effects on women’s moods related to sexual dimorphism in 5-HT availability, energy metabolism, thermoregulation, and sleep-related neurocircuitry repair. Sexual dimorphism of the Trp–melatonin axis may provide some clues, in this respect, for a more thorough understanding of the mental health risks at HA.

## 2 Special topics

### 2.1 sexual dimorphism, 5-HT, and mood disorders

Sex (biological constructs) and gender (social constructs) have to be considered in all adaptive physiological responses (Mauvais-Jarvis et al., 2020; Riveros-Rivera et al., 2022). “Sex” refers to biological characteristics and “gender” to social factors associated with being male or female (Habib and Messing, 2012; Laberge et al., 2020). sexual dimorphism, at least partially, includes the brain structure and function (Choleris et al., 2018; McEwen and Milner, 2017). Estrogen and progesterone receptors have been detected in the cortex, limbic system, hypothalamus, and pituitary gland (Risco, 2010). Early developmental and final permanent effects of the reproductive hormones determine a sex-related brain dimorphism, the latter basically consisting of organizational differences of the neural networks (Littlejohn et al., 2020), their sex-specific genetic architecture presumably resulting from a combination of sex-specific and sex-independent loci (Weiss et al., 2005). Neuropsychiatric disorders, such as post-traumatic stress, autism, and schizophrenia, and general social behavior and sensory processing appear often linked to alterations of the serotonergic and dopaminergic neurotransmitter systems (Lyon et al., 2020). Particularly, 5-HT synthesis represents a key for neuroendocrine disorders (Rahman et al., 2011). 5-HT synthesis occurs in the brain neurons and gut enterochromaffin cells. Peripheral 5-HT contributes to constitute the gut–brain axis. The mean rate of 5-HT synthesis in the brain is 1.5-fold higher in males than in females (Nishizawa et al., 1997). The platelet 5-HT content, on the other hand, is higher in women than in men (Gonzales, 1980; Weiss et al., 2005; Chakraborti et al., 2020). On chronic exposure to hypobaric hypoxia, brain neurotransmitters tend to associate with those in the peripheral blood, possibly because of the blood–brain barrier permeabilization (Zhong et al., 2021). Trp depletion is associated with depression (Cowen, 2008). Abnormal metabolism of 5-HT has also been related to depression (Lu et al., 2017). The lower levels of platelet 5-HT are associated with suicide risk (Giurgiuca et al., 2016). Low GH and low IGF-1 levels have also been associated with major depressive disorder (MDD) and anxiety (Santi et al., 2018; Karachaliou et al., 2021; Wainberg et al., 2021). Increasing brain 5-HT activity in humans elevates plasma levels of prolactin, growth hormone (GH), and adrenocorticotropic hormone (Cowen et al., 1990).

**FIGURE 1:**
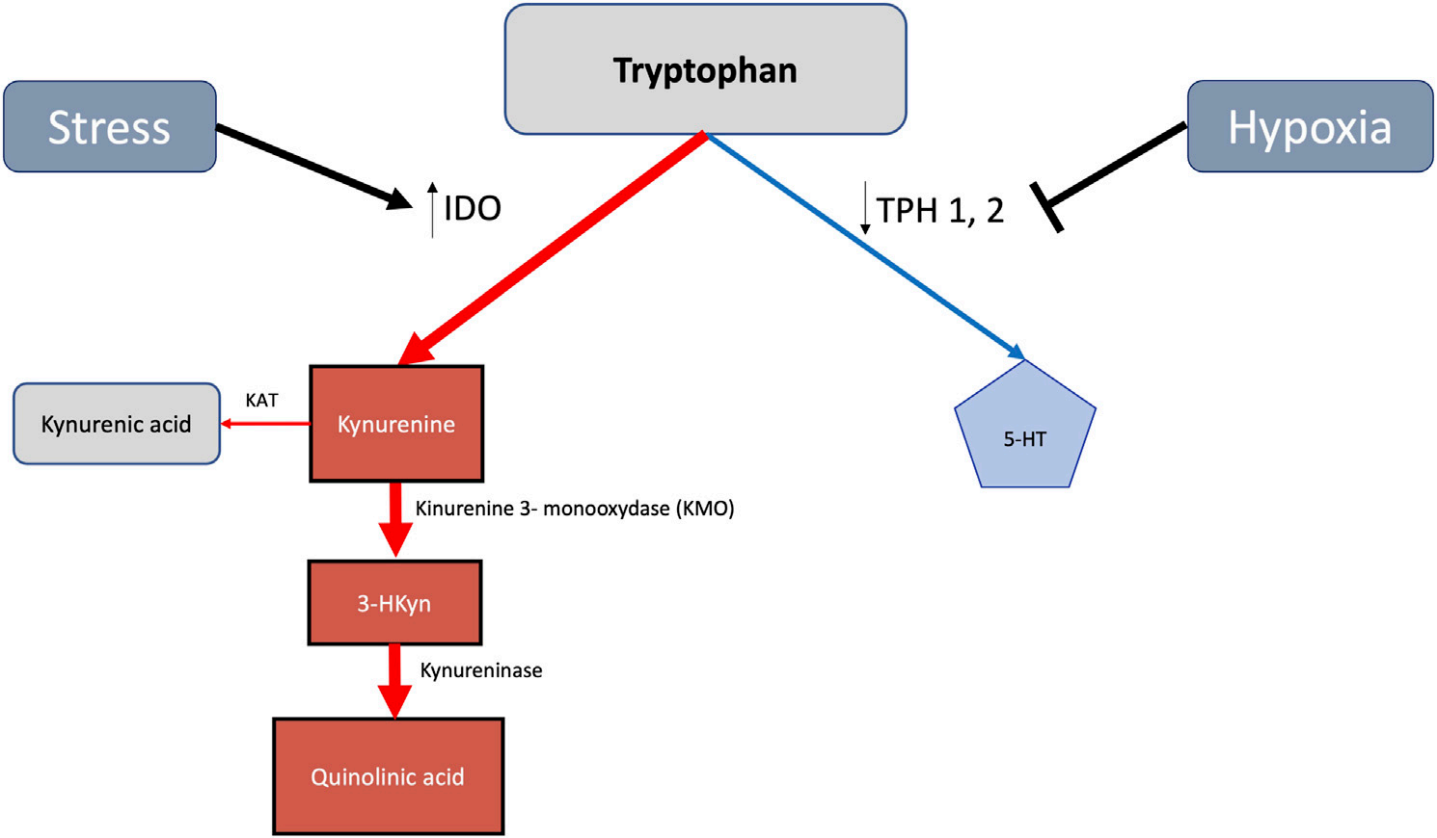
Stress and hypoxia mediated increase of the tryptophan derived metabolic kynurenine 128 pathway in detriment of 5-HT generation. Indoleamine 2,3-dioxygenase (IDO), being upregulated 129 by stress, enhances toxic kynurenine signaling. By reducing tryptophan hidroxylase (TPH) activities, 130 hypoxia concomitantly diminishes 5-HT availability.

Most of body 5-HT is derived from dietary Trp (Figure 1). After its hydroxylation and subsequent decarboxylation, Trp leads to 5-HT, a highly conserved monoamine neurotransmitter involved in behavior and regulation of metabolism (Yabut et al., 2019). Approximately 1% of Trp is converted into 5-HT and other downstream metabolites (Mondanelli and Volpi, 2021). Trp hydroxylases (TPHs), which finally yield 5-hydroxyindoleacetic acid (5-HIAA), have two isoforms: TPH2 in the neurons and TPH1 in the peripheral organs (Schoenichen et al., 2019). Estradiol increases TPH2 activity, thereby enhancing 5-HT synthesis and also 5-HT transporter (SERT) expression (Hernández-Hernández et al., 2019). 5-HT reuptake in the presynaptic neuron through SERT, a membrane 5-HT transporter, terminates neurotransmission (Nagayasu, 2022). The enhancement of SERT function predominantly affecting female mice (Haase et al., 2021) enhances 5-HT clearance, thereby directly reducing 5-HT receptor responsiveness. Female mice overexpressing the 5-HT transporter (SERT + mice) develop pulmonary arterial hypertension, whereas male SERT + mice remain unaffected (White et al., 2011). SERT is also the target of most antidepressants (Bouali et al., 2003). Arylalkylamine-N-acetyltransferase (AANAT) N-acetylates converts 5-HT to N-acetylserotonin, which is subsequently converted by hydroxyindole-O-methyltransferase, thus leading finally to melatonin (N-acetyl-5-methoxytryptamine). Melatonin, increasing during the luteal phase of the menstrual cycle (Cipolla-Neto et al., 2022), is supposed to elicit female aggressive behavior by releasing androgens from adrenal cells (Rendon et al., 2015).

At sea level, anxiety and depression occur twice as often in women than in men (Weissman et al., 1996; 1994; Gater et al., 1998). Depression contributing to drug abuse (Brook et al., 2002) occurs more frequently in females who also appear to be more vulnerable toward addiction (Becker and Chartoff, 2019). Moreover, women are twice more likely than men to develop post-traumatic stress disorder (PTSD), a condition evolving with increasing sympathetic reactivity and decreasing parasympathetic activity, with chronic inflammation also appearing to be involved (Fonkoue et al., 2020). Pro-inflammatory cytokines seem to participate in causing depression. Patients treated with interferon alpha (IFN-α) show reduced levels of Trp, augmented levels of kynurenine (Kyn), and elevated Kyn/Trp ratio activity together with an increase in depressive symptoms (Correia and Vale, 2022). An elevated Trp to kynurenine ratio, conversely, reflects endogenous adaptation to stress (Poeggeler et al., 2022).

Trp hydroxylases (TPHs) require molecular oxygen as a substrate, with their activities potentially being curtailed by hypoxia (Davis, 1975). Hypoxia and stress reduce TPH activity, thereby diminishing 5-HT generation (Young, 2013). The Trp–kynurenine pathway, on the contrary, concomitantly becomes enhanced (Figure 1) and also increases Trp degradation into kynurenine and quinolinic acid (Mohapatra et al., 2021). The latter anxiogenic agents (Figure 1) potentially trigger panic disorder, phobias, and post-traumatic stress (Kim and Jeon, 2018). Indoleamine-2,3-dioxygenase (IDO), a heme enzyme, catalyzes the rate-limiting step in the kynurenine pathway of Trp metabolism (Figure 1). Hypoxia increases IDO generation in dendritic cells (Song et al., 2018). Activating the Trp–kynurenine pathway, IDO may lead to abnormal 5-HT levels in the brain and trigger depressive disorders (Lu et al., 2017). 5-HT blood levels appear to be reduced 1.64 times in post-menopausal women at low altitudes and 1.25 times at HA (Gonzales and Carrillo, 1993). Whole blood 5-HT increases in men native to high altitude but does not change in women (Gonzales, 2014). 5-HT concentrations in the hippocampus, cerebral cortex, cerebrospinal fluid, and plasma decrease on exposure to HA in rats and in the plasma of men (Kious et al., 2018). Severe hypobaric hypoxia reduces rat brain 5-HT levels by approximately 30% (Prioux-Guyonneau et al., 1982). Together with a decrease in 5-HT content in the forebrain and lungs, hypoxia leads to an accumulation of 5-hydroxy-Trp (Izikki et al., 2007). Hypoxia also increases resistance to 5-HT reuptake inhibitors (Kanekar et al., 2018) and blocks the 5-HT1A receptor (Dutta et al., 2022). HA-associated cognitive impairment correlates with concomitant decrements of 5-HT plasma levels (Zhong et al., 2021). Under hypoxic conditions, Trp is also increasingly converted to tryptamine (Mohapatra et al., 2021). Tryptamine resulting from melatonin degradation also causes hallucinations (Malaca et al., 2020). Hypoxia-related enhancement of IDO is at least partially reverted by estrogen (Songtachalert et al., 2018). Hypoxia-induced TPH inhibition is also partially reverted by estrogen (Rahman and Thomas, 2013). An increase in oxygen availability by the continuous positive airway pressure (CPAP), on the other hand, improves sleep patterns in humans and restores plasma 5-HT concentration (Madaeva et al., 2021).

### 2.2 5-HT, REM sleep, and thermoregulation

Sleep-related complaints also appear to be more frequent in women than in men (Krishnan and Collop, 2006; Jehan et al., 2016), eventually becoming even more common with aging (Goel et al., 2005). Hypoxia increases sleep disturbances particularly in women which apparently increases even more when combined with bed rest applied as a quasi-weightlessness condition to simulate spacecraft and/or planetary environments (Morrison et al., 2017; Van Cutsem et al., 2022). Nocturnal oxygen saturation inversely relates to serum melatonin concentration, at least in males (Calderón-Jofré et al., 2021), suggesting that melatonin may also be involved in altitude-associated sleep impairments. Sleep issues are strongly linked to psychiatric disorders (Benca, 1992; Wetter et al., 2008). The network organization of REM sleep seems to be more vulnerable in women than in men (Palagini et al., 2013; Baglioni et al., 2016; Hein et al., 2020). Short REM sleep latency (the interval between sleep onset and the first REM sleep period), an increase in REM sleep duration, and the density of REMs during this state are considered important biological markers of depression (Torterolo et al., 2015). Patients with REM sleep behavior disorder (RBD) tend to develop neurodegenerative diseases (Schenck and Mahowald, 2002; Boeve et al., 2003). Memory processing and learning are thought to occur by selective pruning of synapses (Li et al., 2017). Synapse efficacy, reduced by daily wear, is restored during sleep, thus allowing contextual restraints of the brain to be coordinated and reinforced. Memory consolidation is also implicit for emotion handling (Wagner et al., 2001), the latter, however, being more recently questioned (Lehmann et al., 2016). An adequate REM sleep diminishes the possibility of picture memories becoming excessively intrusive (Werner et al., 2021).

Besides a potential role in memory consolidation (Boyce et al., 2016), REM sleep, although rather passive, appears to be involved in thermoregulation (Kavanau, 1997; Komagata et al., 2019; Cerri and Amici, 2021). Total sleep deprivation and selective REM sleep deprivation induce hypothermia, which even lead to death in rats (Rechtschaffen and Bergmann, 2002). Besides hypothermia, syndromes induced by sustained REM sleep deprivation include a notorious increase in food intake and weight loss (Rechtschaffen and Bergmann, 2002). Interestingly, acute exposure to HA often leads to a very similar pattern. Appropriately sustaining thermogenesis should be particularly important at HA, an environment that lends to sleep deficiency and also potential exposure to cold. Women have been reported to shiver less in cold stress than do men (Iyoho et al., 2017). Cold-induced thermogenesis, on the other hand, has been reported to be higher in women than in men, a condition independently associated with estradiol (Herz et al., 2021). Chronic stress, as supposed to prevail on exposure to chronic hypoxia, disrupts the normal pattern of daily body temperature by decreasing the presence of 5-HT in the medial preoptic area (mPOA). 5-HT agonist injected into the mPOA, on the contrary, restores normal body temperature cycling (Natarajan et al., 2015). The mPOA represents a sex dimorphic structure (Wei et al., 2018) that also coordinates emotions and behavior (Zhang et al., 2021). By tightly interacting with the melanin-concentrating hormone (MCH) in the median raphe nucleus, 5-HT is involved in REM sleep and mood control (Pascovich et al., 2021). Mediated by reactive oxygen species (ROS) generated in the hypothalamus through an NADPH oxidase–dependent pathway, 5-HT acts as an anorexigenic agent (Fang et al., 2013), in contrast to the orexigenic MCH (Izawa et al., 2019), where the latter reduces energy expenditure (Zheng et al., 2005) and decreases body temperature (Zheng et al., 2016; Ishihara et al., 2021). The greatest firing rate of MCHergic neurons occurs during REM sleep, with the optogenetic stimulation of the latter neurons inducing sleep (Torterolo et al., 2015). Neurons producing MCH lead REM sleep to promote memory loss. The fine-tuned REM sleep expression and regulation, moreover, require a strong interplay between 5-HT systems and hypocretin/orexin (Seifinejad et al., 2020). The orexin/orexin receptor system appears to constitute an anxiogenic organization, also being more dominant in women than in men (Freeman and Aston-Jones, 2020). Orexin levels in the cerebrospinal fluid have been found to be elevated in patients with anxiety disorder (Bi et al., 2022). 5-HT inhibits orexin neurons (Muraki et al., 2004; Saito et al., 2018), but its availability is diminished at HA.

**FIGURE 2:**
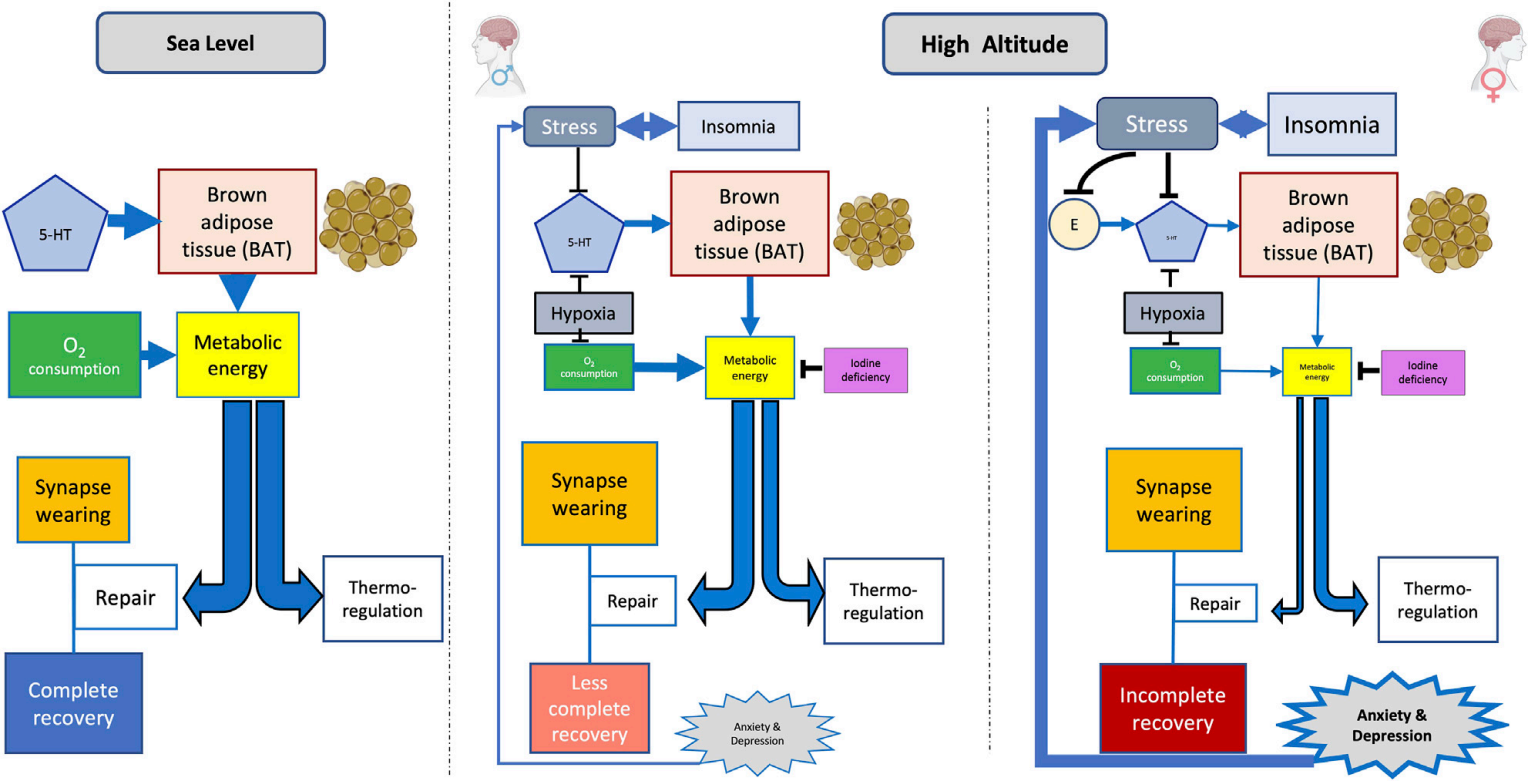
Hypothetical sex-related mental disorder on exposure to high altitude (HA). Stress and hypoxia decrease the availability of 5-HT at HA; brain synthesis of the latter already being lower in women than in men. Moreover, estrogen (E)-mediated support of 5-HT is blocked by stress. The lack of 5-HT diminishes brown adipose tissue (BAT)–dependent heat generation. Metabolic energy already being reduced by hypoxia, and possibly also by goiter, which may consequently generate a tradeoff in order to prioritize the energy requirements of thermoregulation, possibly at the expense of synapse repair. Memory consolidation thereby being interfered could allow mood changes to surge; the latter, in turn, additionally enhancing stress and insomnia. A vicious circle may thus be installed, potentially triggering the generation of mental disorders. Line thickness indicates the magnitude of effects.

REM sleep, thought to support CNS maturation and optimization (Chen et al., 2022), tends to decrease on exposure to cold (Komagata et al., 2019). 5-HT neurons lead BAT to enhance heat generation by increasing the sympathetic drive (Yabut et al., 2019). By activating adrenergic receptors in BAT, norepinephrine stimulates triglycerides stored in white adipose tissue (WAT) to hydrolyze into free fatty acids (FFAs). The latter activates BAT-specific uncoupling protein 1 (UCP1) in the inner mitochondrial membrane. UCP1 activation generates heat by uncoupling oxidative phosphorylation from ATP synthesis. The mitochondrial inner membrane uncoupling protein 2 (UCP2), an anion carrier, senses changes in oxygen levels. The upregulation of UCP2 is essential for metabolic adaptation in hypoxic environments (Wu et al., 2021; Rigaud et al., 2022). UCP2, moreover, plays an essential role in the development of cognitive ability and resistance to anxiety (Wang et al., 2014). The blockade of UCP2, on the other hand, produces cognitive impairment and anxiety in young mice. Serotoninergic fluoxetine in rats overfed during the suckling period improves their mitochondrial function and oxidative balance and, moreover, activates signaling pathways that increase mitochondrial biogenesis and metabolism (Zhong et al., 2021). Heat generated by BAT may contribute to supporting the core temperature during REM sleep. BAT appears to be more developed in females than in males (Keuper and Jastroch, 2021) but requires 5-HT to be fully activated. In a cold and hypoxic environment such as in HA, that is additionally devoid of 5-HT, a lack of energy availability may lead to a resource allocation tradeoff favoring the maintenance of body temperature at the expense of synapse repairs (Figure 2). The failure of sleep-related synapse reinforcement implies an accumulation of rather degraded circuitry and possibly underlying mood disorders (Kavanau, 2000).

### 2.3 5-HT and monoamino oxidases

Bound to the outer mitochondrial membrane, monoamino oxidases (MAOs) are ROS generators. Catechol-O-methyl transferase and MAOs appear to be less affected by HA than are tyrosine and TPHs, but their activities always tend to decrease (Vaccari et al., 1978). MAO activity in the cerebral cortex on exposure to HA is occasionally been found to be somewhat lower in females than in males (Vaccari et al., 1978). Corresponding with a possibly higher dopaminergic activity at HA, serum prolactin levels appear to be diminished both in women and men living in the Peruvian highlands (Gonzales et al., 1992; Gonzales and Carrillo, 1993; Solís et al., 2011). The plasma dopamine-beta-hydroxylase (E.C. 1.14.17.1) activity increases in rats acutely exposed to HA; this increase is significantly more notorious in females than in males (Koudelová and Mourek, 1990). Dopamine and noradrenaline turnovers accordingly appear to be higher in the female carotid body and brainstem noradrenergic cell groups than in the corresponding male structures in rats reared at an altitude of 3,000 m. Orchidectomy increases dopamine and noradrenaline turnover in the carotid body and brainstem noradrenergic cell groups and ovariectomy decreases it (Pequignot et al., 1997). Peripheral plasma DOPA, dopamine, 5-HT, 5-HIAA, and glutamate levels are associated with brain neurotransmitter levels after chronic HA exposure in rats. Chronic HA exposure decreases dopamine but increases DOPA levels in the human plasma. 5-HT, on the other hand, decreases and 5-HIAA levels increases in humans on chronic HA exposure (Zhong et al., 2021).

### 2.4 5-HT, thyroid, and gonadal hormones

The Trp–5-HT axis also links with thyroid function, with both pathways influencing mood (Bauer et al., 2002) and thermoregulation. Thyroid replacement therapy reverses the reduced responsiveness to 5-HT in hypothyroid patients and represents an effective adjunct for treatment of affective disorders (Bauer et al., 2002). Selective 5-HT reuptake inhibitors, on the other hand, seem to decrease thyroid function (Caye et al., 2020). Iodine deficiency in mountainous regions and corresponding hypothyroidism must be considered in this respect (Figure 2). Goiter being more prevalent in iodine-deficient areas, particularly affects females (Pretell et al., 1969; Malboosbaf et al., 2013).

HA exposure tends to suppress the gonadal axis (von Wolff et al., 2018). Estrogens upregulate 5-HT2A receptors in the brain (Cyr et al., 1998). Their effect on mood may be also mediated through influences on 5-HT2A and 5-HT2C receptor expressions (Birzniece et al., 2002). Estrogen receptor 2 (ESR2) mediates estrogen effects on TH1 and TH2 expressions (Hiroi and Handa, 2013). Estradiol acting on estrogen receptor β (ERβ) inhibits 5-HT reuptake by downregulating the gene expression of SERT in the neurons and astrocytes. Estradiol may thus act as an antidepressant by increasing 5-HT availability (Gu et al., 2022). Estrogen treatment alone or in combination with testosterone decreases SERT in several cortical, limbic, and striatal regions of surgically ovariectomized women (Jovanovic et al., 2015). Low estrogenic activity, on the other hand, may result in low 5-HT availability. Cognitive performance improves in the luteal phase when compared with the follicular phase of the menstrual cycle (Grant et al., 2021). Premenstrual syndrome (PMS) mostly expresses as an increase in irritability and fatigue during the late luteal or premenstrual phase and finally disappears when menses initiates (Pearlstein et al., 2005). Selective 5-HT reuptake inhibitors (SSRIs), applied along the luteal phase, are effective in treating premenstrual syndrome (Yonkers and Simoni, 2018; Carlini and Deligiannidis, 2021). 5-HT2A receptors regulate responses to stress (Czesak et al., 2012). On the other hand, elevated expressions of the 5-HT1A receptor gene and reduced raphe 5-HT levels appear to be involved in the generation of major depressive disorder (MDD) and other mood changes. 5-HT1A receptors in the dorsal raphe nucleus appear to be upregulated during the depressive state (Li et al., 2020).

Remarkably, estrogen levels during pregnancy appear to be higher in Andean residents than in European residents at HA (Charles et al., 2014). In ovulating women, the luteal phase increase of serum estradiol occurred earlier at sea level than at HA (Escudero et al., 1996). By modulating the expression of the 5-HT receptor—*5-HT2BR—*estrogens decrease cardiomyocyte death that occurs in response to hypoxia/re-oxygenation injury. Glucocorticoids, on the other hand, prevent 5-HT–mediated estrogen cardioprotection, exacerbating the size of the infarct areas in myocardial infarction (Dhaibar et al., 2021). 5-HT protection by estrogens, being cancelled by glucocorticoids, evidence the deleterious effects that stress may have on women’s well-being, particularly under hypoxic conditions.

## 3 Discussion and conclusion

A surveillance of human activities at HA increasingly requires sexual (and gender)-dimorphism to be considered in tolerance to hypobaric hypoxia. HA exposure decreases slow-wave sleep (SWS), REM sleep, sleep efficiency, and the total sleep time (Weil, 2004). Hypoxia affects REM sleep (Riveros-Rivera et al., 2022), the organization level of the latter having been shown to be more vulnerable in women than in men (Palagini et al., 2013; Baglioni et al., 2016; Hein et al., 2020). REM sleep disruption may affect memory consolidation, which includes the emotional state—mood alterations, anger, and higher fatigue scores thus arise (Stavrou et al., 2018). Besides affecting mood stability, HA exposure decreases attention, visual and working memory, concentration, executive functions, inhibitory control, and the speed of mental processing (Aquino Lemos et al., 2012; Bloch et al., 2015). Involving the Trp–melatonin axis, hypoxia also reduces 5-HT synthesis (Young, 2013). The failure of BAT activation due to lack of 5-HT (Yabut et al., 2019) additionally favors the development of hypothermia.

Figure 2 summarizes the postulated sexual dimorphism of 5-HT availability at HA and the related pathogenesis of mood disorders. Hypoxia affects metabolic energy generation. The shortage of metabolic energy at HA generates tradeoffs between different energy destinations, which include thermoregulation and sleep-related neural circuitry repair. Hypoxia-induced insomnia, which includes REM sleep deprivation, concomitantly with the lack of 5-HT, may lead to energy being preferentially diverted into thermoregulation in women, perhaps also at the expense of adequate synapse restoration. Defective neurocircuitry repair may affect the consolidation of memory such as the emotional state, with mood disorders thus emerging. Stress and insomnia are, thereby additionally enhanced, in turn reinforcing already prevailing mood disorders. A vicious circle may thus evolve, possibly affecting mental health. Hormonal changes during the menstrual cycle, and certainly also during the life cycle, as well as enhanced stress susceptibility, underscore the possibility of 5-HT–related mood alterations in women exposed to HA. Besides caring for adequate oxygen supply, maintenance of adequate body temperature, stress mitigation, and close observation of menstrual cycles, trials with selective 5-HT reuptake inhibitors may also be envisaged for health, well-being, and safety of women sojourning at HA.
